# Can PRP Enhance Hamstring Recovery Post-ACL Reconstruction? Retrospective Insights from Non-Professional Athletes

**DOI:** 10.3390/jcm14238266

**Published:** 2025-11-21

**Authors:** Roxana Mihaela Munteanu, Andrei Marian Feier, Bogdan Voicu, Arpad Solyom, Pia Simona Făgăraș, Tudor Sorin Pop

**Affiliations:** 1 Doctoral School, George Emil Palade University of Medicine, Pharmacy, Science, and Technology of Targu Mures, 540142 Targu Mures, Romania; roxana-mihaela.munteanu@umfst.ro; 2 Department M2 Functional and Complementary Sciences, Epidemiology, Ethics and Socio-Human Sciences, George Emil Palade University of Medicine, Pharmacy, Science, and Technology of Targu Mures, 540139 Targu Mures, Romania; drbogdanv@gmail.com (B.V.);; 3 OKF Medical Center, 540139 Targu Mures, Romania; arpad.solyom@umfst.ro; 4 Department M4 Clinical Sciences, Orthopedics and Traumatology I, George Emil Palade University of Medicine, Pharmacy, Science, and Technology of Targu Mures, 540139 Targu Mures, Romania; tudor.pop@umfst.ro; 5 Department of Orthopaedics and Traumatology, Clinical County Hospital of Mureș, 540139 Targu Mures, Romania; 6 Department M3 Clinical Sciences, Orthopedics and Traumatology II, George Emil Palade University of Medicine, Pharmacy, Science, and Technology of Targu Mures, 540139 Targu Mures, Romania

**Keywords:** anterior cruciate ligament reconstruction, hamstring recovery, platelet-rich plasma, muscle strength, rehabilitation

## Abstract

**Background and Objectives**: Hamstring strength deficits are common after ACL reconstruction and may impair stability and increase reinjury risk. Despite structured rehabilitation, early postoperative asymmetries frequently persist. Platelet-rich plasma (PRP) has emerged as a potential biologic adjunct that may enhance tissue healing and facilitate muscle strength recovery. This study aimed to evaluate the efficacy of intra-articular PRP injections on hamstring strength restoration in non-professional athletes undergoing ACLR. **Materials and Methods**: This retrospective cohort study analyzed 68 non-professional athletes who underwent primary ACLR between 2020 and 2024. Participants followed a standardized, phase-appropriate rehabilitation program and were allocated to either a PRP group (n = 34; five intra-articular PRP injections every 4 weeks starting at week 4 postoperatively) or a control group (n = 34; rehabilitation alone). Data were analyzed with linear mixed-effects models to examine group, time, and group-by-time interactions. **Results**: Both groups improved over time, but PRP influenced faster hamstring strength recovery and greater symmetry. At 20 weeks, the PRP group achieved higher concentric strength (15.6 ± 4.3 vs. 13.6 ± 3.5; *p* = 0.040) and markedly reduced asymmetry (isometric: –1.1 vs. 0.6; *p* < 0.001; concentric: –0.6 vs. 1.9; *p* < 0.001). **Conclusions**: The addition of serial PRP injections to a structured rehabilitation protocol after ACLR influenced faster hamstring strength recovery and improved interlimb symmetry compared to rehabilitation alone. These findings suggest that PRP may serve as a potential biologic adjunct to optimize postoperative outcomes and facilitate a safer return to sport in non-professional athletes.

## 1. Introduction

Anterior cruciate ligament reconstruction (ACLR) is widely performed in active individuals, yet postoperative deficits in hamstring strength remain a persistent challenge, particularly when hamstring autografts are used [1,2,3]. Hamstring function plays an important role in lower-limb stability, gait control, and athletic performance, and incomplete recovery affects biomechanics, reduced dynamic stability, and an increased risk of reinjury during return-to-sport activities [4,5]. Despite structured rehabilitation protocols, many patients experience prolonged or incomplete restoration of knee flexor strength, emphasizing the need for strategies that may optimize postoperative muscle recovery [6].

Restoration of hamstring strength is clinically relevant not only for performance but also for joint protection [4]. The hamstrings act as dynamic synergists of the ACL by limiting anterior tibial translation, and strength deficits—especially at deeper flexion angles—may persist long after surgery [7]. Current rehabilitation approaches target multiple strength qualities, including maximal, eccentric, and multiplanar capacity [4], yet asymmetries frequently remain during the early return-to-sport phase [8]. These deficits can affect the quadriceps-to-hamstring (Q/H) strength ratio, an important marker of neuromuscular readiness and reinjury risk [9].

Given these challenges, biologic augmentation has been explored as a potential adjunct to enhance postoperative recovery [10]. Platelet-rich plasma (PRP) is an autologous concentrate rich in growth factors such as PDGF, TGF-β, VEGF, and IGF-I, ref. [11] which may modulate inflammation, support tissue remodeling, and influence the intra-articular environment after ACLR [12].

Although PRP has demonstrated potential benefits in conditions such as tendinopathy [13], rotator cuff repair [14], and osteoarthritis [15], its role in ACL reconstruction remains unclear. Evidence across studies is heterogeneous: some report improvements in pain or graft maturation [16], whereas others—including recent randomized clinical trials—find no significant clinical benefit in postoperative function or knee symptoms [17].

Importantly, few studies have examined whether intra-articular PRP may influence postoperative hamstring muscle recovery specifically [17,18].

While PRP has been proposed to modulate neuromuscular inhibition and periarticular tissue healing, its effect on objective strength outcomes after ACLR is not well established, and data regarding interlimb strength symmetry remain limited [16]. Thus, the clinical relevance of PRP in supporting hamstring strength restoration during rehabilitation has not been adequately investigated [19].

To address this gap, the present retrospective cohort study evaluated whether serial intra-articular PRP injections, administered in conjunction with a standardized postoperative rehabilitation program, were associated with improved hamstring strength recovery and interlimb symmetry in non-professional athletes following ACLR.

## 2. Materials and Methods

### 2.1. Study Design

This retrospective cohort study included 68 subjects who underwent ACLR between 2020 and 2024. Participants were divided into two groups: a treatment group that followed a standardized rehabilitation protocol and received PRP infiltrations, and a control group that followed the same protocol without PRP administration. Because this was a retrospective study, group allocation was not randomized. Patients were assigned to the PRP or control group based on the treatment they had already received as part of routine clinical practice. All eligible patients were enrolled consecutively according to the order of presentation, ensuring naturalistic sampling without investigator influence. Controls were drawn from the same clinical population and met the same inclusion criteria as the PRP group. The inclusion criteria were patients who underwent primary ACL reconstruction between 2020 and 2024, non-professional (amateur) athletes engaged in regular physical activity, no concomitant ligament injuries (isolated ACL tear). Exclusion criteria included the presence of cardiovascular or musculoskeletal disorders, use of corticosteroid therapy, or occurrence of postoperative injuries during the rehabilitation period. The exclusion criteria were the missing data on muscle strength. Although strict matching was not performed, the two groups were comparable in terms of age, sex, graft type, and baseline hamstring strength. Collected data included patient age, sex, body mass index (BMI), graft type, general health status, and current medication.

A total of 82 patients who underwent primary ACL reconstruction were screened for eligibility. Fourteen patients were excluded due to incomplete rehabilitation data (n = 8), concomitant meniscal repair requiring restricted loading (n = 4), or missing follow-up strength measurements (n = 2). The remaining 68 patients were included in the analysis: 34 who received PRP injections in addition to rehabilitation and 34 who underwent standard rehabilitation only.

**Figure 1:**
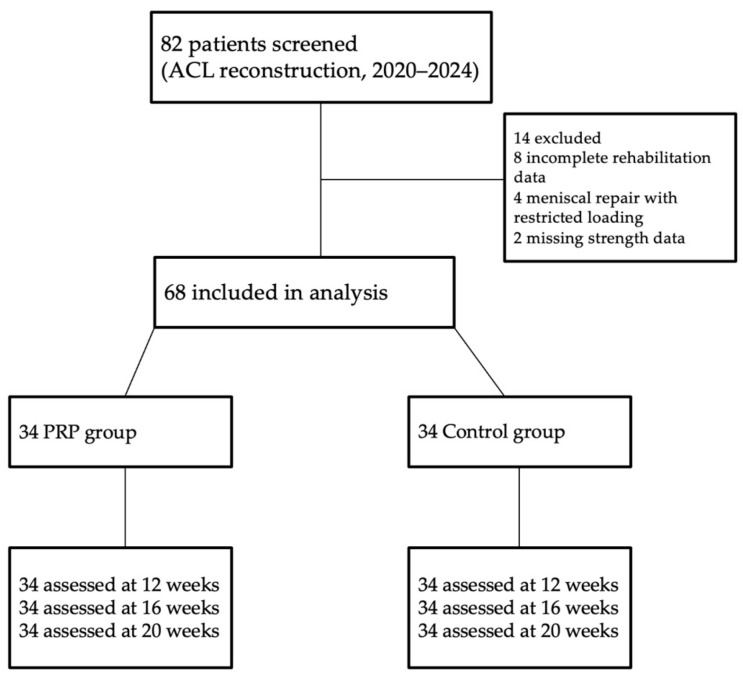
STROBE flow diagram of patient selection and follow-up. Flow of participants from initial screening through inclusion, allocation to PRP or control groups, and assessment at each follow-up point, including reasons for exclusion and missing data.

A detailed flow diagram illustrating patient inclusion, exclusion, and follow-up is provided in Figure 1.

This retrospective cohort study included consecutively enrolled non-professional athletes who underwent ACL reconstruction at OKF Medical Center Târgu Mures. All eligible patients meeting the inclusion and exclusion criteria were analyzed.

This retrospective study was approved by the Ethics Committee for Scientific Research of the George Emil Palade University of Medicine, Pharmacy, Science and Technology of Târgu Mureș (approval nr. 3562/20 January 2025).

### 2.2. Rehabilitation Protocol

All participants completed the same standardized rehabilitation program consisting of 3–4 supervised sessions per week. The intervention included progressive phases targeting restoration of knee mobility, neuromuscular activation, progressive strengthening, proprioceptive and balance training, plyometric exercises, activity-specific and sport-specific drills. Progression was based on predefined clinical criteria (pain < 3/10, full extension, adequate quadriceps control).

Rehabilitation sessions were delivered by licensed physiotherapists. Therapists were not blinded to PRP allocation. Rehabilitation adherence was 100%, with all subjects completing the prescribed sessions.

### 2.3. PRP Intervention

**Figure 2:**
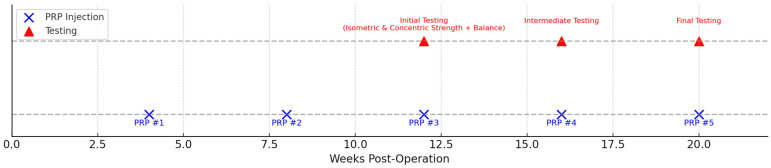
Timeline of PRP injections and outcome assessments following ACLR. Five intra-articular PRP injections were administered at approximately 4, 8, 12, 16, and 20 weeks postoperatively (blue “×”). Functional testing sessions (red triangles) were scheduled at three key phases: Initial Testing (around week 10; isometric and concentric quadriceps and hamstrings strength combined with static balance assessment), Intermediate Testing (around week 15; progressive strength and functional control evaluation), and Final Testing (around week 20; comprehensive return-to-sport assessment). This timeline illustrates the structured integration of biologic augmentation and objective performance monitoring during postoperative rehabilitation.

The experimental group received five intra-articular injections of autologous leukocyte-poor platelet-rich plasma (PRP). Injections were administered at weeks 4, 8, 12, 16, and 20 postoperatively by the orthopedic surgeon. Each injection consisted of 5 mL of P-PRP. The schedule of PRP injections and functional testing sessions is illustrated in Figure 2. 

PRP was prepared using the double-spin protocol described by Dhurat et al. [11]. Centrifugation was performed using a specialized centrifuge (XC SPINPLUS, Shanghai, China) in a two-step (double-spin) process. 

A total of 30 mL venous blood was drawn into citrate-dextrose tubes, centrifuged at 1200 rpm for 10 min, followed by a second spin at 3000 rpm for 10 min. This yielded 3–5 mL of leukocyte-poor PRP, injected intra-articularly without exogenous activation.

The use of multiple PRP doses was supported by literature indicating that repeated administrations enhance cumulative biological effects. Tao et al. [20] demonstrated superior outcomes with three sequential injections, while Corsini et al. [21] highlighted the importance of achieving sufficient cumulative platelet dosing for optimal anti-inflammatory and reparative activity.

### 2.4. Outcome Measures

Primary outcomes included: Maximal isometric hamstring strength, Maximal concentric hamstring strength, Limb strength asymmetry, calculated as:

Asymmetry (%) = [(Injured limb − Healthy limb)/Healthy limb] × 100.

Positive values indicate greater strength in the injured limb, whereas negative values indicate a deficit compared to the healthy limb. These parameters represent surrogate indicators of early functional recovery.

Evaluations were performed at 12, 16, and 20 weeks postoperatively, corresponding to key rehabilitation phases: 12 weeks: early strengthening following graft integration, 16 weeks: advanced neuromuscular training phase, 20 weeks: late-phase functional progression. This timing is supported by prior evidence by San Jose et al., showing persistent hamstring deficits during the first 3–6 postoperative months [22].

### 2.5. Strength Testing Procedures

Strength assessments were performed using the Kineto Intelligent Load rehabilitation robot. Participants completed a standardized warm-up consisting of 10 min cycling at moderate intensity, open- and closed-chain activation exercises, hamstring-specific drills: leg curls, Romanian deadlifts, hip bridges.

For the Isometric Max Strength Balance Test, the subject was placed in a standing position in front of the device, with ankle cuffs connecting them to the machine. The subject was instructed to perform a knee flexion up to 90 degrees and to hold the position for 5 s, ensuring they exerted maximal force to maintain the flexion. The test was performed once on both the affected and unaffected limb.

For the Concentric Max Strength Balance Test, the subject was also in a standing position, with ankle cuffs, in front of the device, and was asked to perform 5 knee flexions, focusing on the lifting phase, thus emphasizing concentric contraction, using both the affected and unaffected limb.

All evaluations were supervised by the same physiotherapist to ensure consistent technique and avoid compensatory movements.

### 2.6. Statistical Analysis

Descriptive statistics were calculated for all demographic, clinical, and outcome variables. Continuous variables are reported as mean ± standard deviation, and categorical variables as counts and percentages. Due to missing data for BMI this parameter was excluded from statistical analysis. Complete case analysis was performed using linear mixed-effects models fitted by Restricted Maximum Likelihood (REML) estimation to account for the longitudinal study design and repeated measures within participants. Patient-specific random intercepts and slopes for time were included to model individual variability in baseline strength and recovery trajectories. Fixed effects included treatment group, time, age, sex, and graft type (hamstring vs. patellar tendon). Primary outcomes were isometric and concentric hamstring strength of the affected limb, while secondary outcomes examined strength asymmetry between healthy and affected limbs. Group-by-time interaction terms were included to test differential recovery rates between treatment arms. Model assumptions were verified through residual diagnostics, and all models achieved successful convergence. Statistical significance was set at α = 0.05, and analyses were conducted using Python’s statsmodels package (version 0.14.0). Between-group comparisons at each time point were performed using independent *t*-tests with pooled standard deviations; *p*-values were annotated as ns (*p* ≥ 0.05), * (*p* < 0.05), ** (*p* < 0.01), or *** (*p* < 0.001). Since this is a retrospective study, no a priori sample size calculation was conducted, but a post hoc power analysis was performed with G*Power software (University of Düsseldorf, Düsseldorf, Germany, version 3.1.9.7.). The post hoc calculated power (1 − β) was 0.95 for the REML analysis comparing hamstring force (primary outcome) recovery with an effect size d = 0.41 (α = 0.05, two-tailed), measured across 3 timepoints in two groups (n = 34 each). This showed a strong statistical power for detecting group differences caused by PRP intervention. Effect sizes (Cohen’s d) were calculated for final assessments to quantify between-group differences as negligible (|d| < 0.2), small (0.2–0.5), medium (0.5–0.8), or large (>0.8).

## 3. Results

The study population comprised 68 participants (50 males, 18 females; age range 18–54 years) with predominantly hamstring autografts (n = 61, 89.7%). The demographic and baseline characteristics of the participants were analyzed (Table 1), and no statistically significant differences were found between the PRP-treated and control groups regarding age, sex distribution, body mass index, or graft source. Both groups were therefore considered homogeneous and comparable for subsequent analyses. 

All patients were monitored clinically after each PRP injection and during routine rehabilitation visits. No cases of infection, synovitis, or significant joint effusion were observed following any of the five intra-articular PRP injections. Four patients (11.8%) reported mild, transient post-injection pain or swelling lasting less than 48 h, which resolved without medical intervention. No procedure-related complications were recorded.

Linear mixed-effects analysis revealed significant time effects across all models (*p* < 0.001), indicating overall recovery in both treatment groups. Crucially, significant group-by-time interactions were observed for both isometric and concentric strength measures (*p* < 0.001), demonstrating superior recovery rates in the experimental group. The experimental group showed 1.34 units (95% CI: 0.90–1.78) faster isometric strength recovery and 1.61 units (95% CI: 1.15–2.07) faster concentric strength recovery per time point compared to controls. Analysis of strength asymmetry revealed significant group differences, with the experimental group achieving better limb symmetry (isometric asymmetry: β = −0.54, *p* = 0.027; concentric asymmetry: β = −0.65, *p* = 0.053). Male participants consistently demonstrated higher absolute strength values across all measures (effect size: 2.0–5.0 units, *p* < 0.001), while age and graft type did not affect the outcomes (all *p* > 0.05). The affected limb strength improved by 2.3–3.0 units per time point, while strength asymmetry decreased by 1.9–2.4 units per time point, indicating successful rehabilitation progress. All mixed-effects models converged successfully with log-likelihood values ranging from −350.4 to −392.9, and residual diagnostics confirmed appropriate model assumptions.

**Figure 3:**
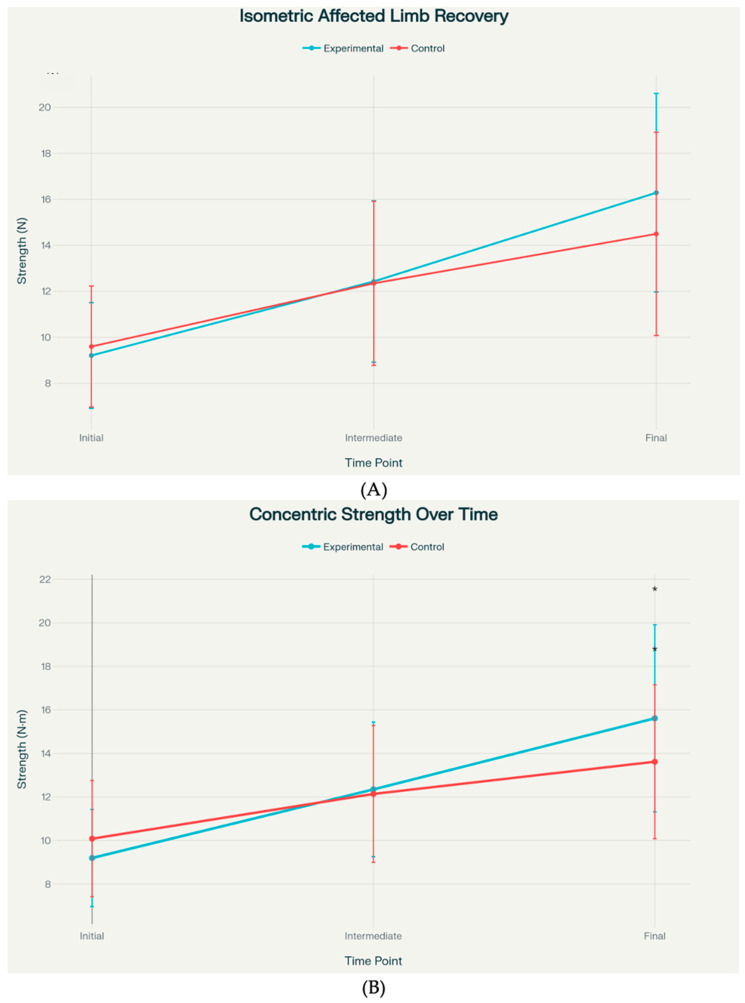
Recovery of hamstring strength in the affected limb over time. (**A**) Isometric strength measurements at initial, intermediate, and final assessments (**B**) Concentric strength measurements at initial, intermediate, and final assessments. Data presented as mean ± standard deviation (n = 34 per group). Statistical comparisons between groups at each time point: ns = non-significant, * *p* < 0.05.

Statistical analysis revealed that concentric strength showed a statistically significant between-group difference at the final assessment, with the experimental group achieving 15.62 ± 4.30 units versus 13.62 ± 3.54 units in the control group (*p* = 0.040). Isometric strength exhibited a strong trend toward significance at the final time point, with means of 16.29 ± 4.32 versus 14.50 ± 4.42 (*p* = 0.097) (Figure 3). Effect sizes for these primary outcomes ranged from small to medium (Cohen’s d = 0.41–0.51).

**Figure 4:**
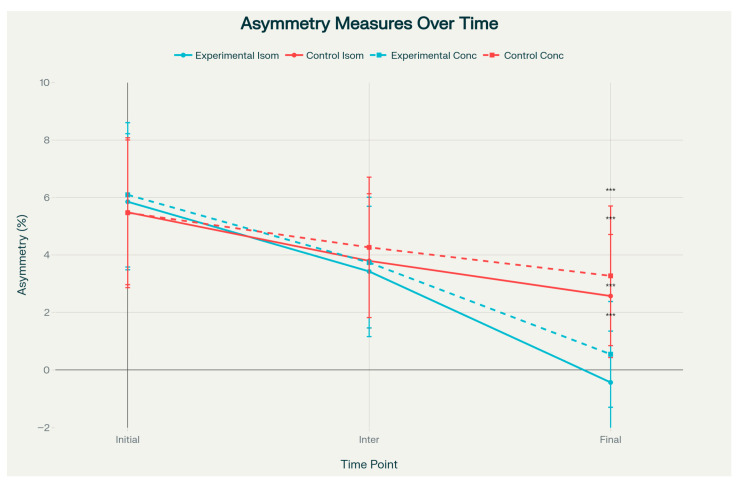
Recovery of strength symmetry between limbs over time. Isometric asymmetry is represented by solid lines, and concentric asymmetry by dashed lines. The control group is shown in red, and the PRP group in blue. Lower values indicate better interlimb symmetry. Data are presented as mean ± SD. The PRP group demonstrated statistically significant but moderate improvements in symmetry at the final assessment for both isometric (*p* < 0.001) and concentric (*p* < 0.001) measures. Negative values in the PRP group indicate that the operated limb achieved strength levels slightly exceeding those of the contralateral limb. *** indicates a statistically significant difference between groups (*p* < 0.0002).

**Figure 5:**
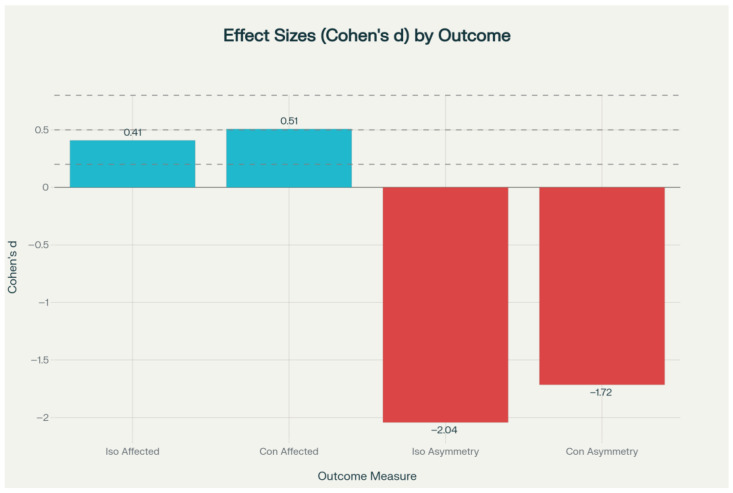
Effect sizes (Cohen’s d) comparing experimental vs. control groups at final assessment. Primary outcomes (affected limb strength) showed small to medium effect sizes, while secondary outcomes (asymmetry measures) demonstrated large effect sizes favoring the experimental intervention. Reference lines indicate effect size thresholds: small (0.2), medium (0.5), and large (0.8).

However, both isometric and concentric asymmetry measures demonstrated statistically significant group differences at the final assessment (*p* < 0.001). The PRP group showed near-zero or slightly negative asymmetry values—indicating that the operated limb approached or marginally exceeded the contralateral limb—with isometric asymmetry of −1.13 ± 0.96 versus 0.64 ± 0.76, and concentric asymmetry of −0.57 ± 1.08 versus 1.93 ± 1.75 (Figure 3). These asymmetry outcomes were associated with large effect sizes (Cohen’s d = −1.72 to −2.04) (Figure 4). The effect size analysis demonstrated small to medium differences in affected limb strength, while asymmetry outcomes showed large effect sizes in the experimental group (Figure 5).

## 4. Discussion

### 4.1. Main Findings

The main finding of this study was that the addition of PRP to a standardized postoperative rehabilitation program influenced statistically significant but modest improvements in hamstring strength recovery and interlimb symmetry in non-professional athletes after ACLR. Participants receiving PRP showed faster strength gains and a more complete restoration of symmetry compared with those undergoing rehabilitation alone, independent of age, sex, or graft type.

While our findings suggest that PRP influences hamstring strength recovery, the effect size was modest, and the results should be interpreted as exploratory. Further prospective, randomized studies are needed to confirm these observations.

Given the retrospective nature of this study, causality cannot be established, and the observed effects should be interpreted with caution.

### 4.2. Comparison with Existing Literature

Persistent hamstring strength deficits after ACLR are well documented. A previous review, by Thomas et al. [2] have reported that residual hamstring strength deficits ACLR can persist in 9–27% of patients even three years postoperatively. In contrast, our PRP-treated cohort achieved near-complete restoration of hamstring strength and interlimb symmetry within 20 weeks, suggesting that adjunctive biologic stimulation may accelerate muscle recovery and potentially reduce the long-term persistence of strength deficits described in the literature.

Recent longitudinal work by Datta et al. [23] demonstrated that hamstring strength and functional parameters improve progressively up to 18 months after ACLR with semitendinosus–gracilis autografts, reflecting the natural, gradual course of recovery. In our cohort, the addition of platelet-rich plasma to a standardized rehabilitation program appeared to accelerate this trajectory, allowing patients to achieve near-complete hamstring strength restoration and interlimb symmetry within 20 weeks. These findings suggest that biologic augmentation may shorten the otherwise prolonged recovery window described in conventional rehabilitation pathways.

Desouza et al. [24] conducted a randomized controlled trial on grade 2 hamstring injuries and demonstrated that PRP can accelerate return-to-sport time and promote faster tissue healing in acute muscle damage. Even though our investigation focused on post-ACLR hamstring recovery rather than acute injuries, the accelerated restoration of strength and improved limb symmetry observed in our PRP-treated group parallels the enhanced healing reported in traumatic hamstring tears, supporting the notion that PRP may facilitate muscle regeneration and functional recovery across different clinical contexts.

While some studies have suggested potential benefits of intra-articular PRP administration on tissue healing or early strength recovery after ACL reconstruction, several systematic reviews and retrospective analyses have failed to demonstrate consistent or clinically meaningful effects. For instance, a comprehensive meta-analysis on the clinical use of PRP in knee disorders concluded that, among the available evidence, ACL reconstruction represents the only area showing no significant differences between PRP-treated and control groups, indicating that PRP has not yet been proven beneficial in this context [18]. These contrasting results highlight the substantial heterogeneity in PRP preparation methods, platelet and leukocyte concentrations, and injection protocols, which may account for the variability in clinical outcomes across studies.

Although a recent randomized controlled trial investigating intra-articular PRP after ACLR reported no significant improvements in patient-reported knee symptoms or overall joint function at 12 months [17], our study specifically targeted objective hamstring strength recovery and demonstrated meaningful benefits in both strength restoration and limb symmetry. This suggests that PRP’s impact may be more readily detectable in direct muscle performance metrics than in broader subjective functional scores.

### 4.3. Potential Mechanism

Recent evidence suggests that intra-articular PRP may influence not only graft healing and joint homeostasis but also neuromuscular recovery through its biological effects on the intra-articular environment. Gill et al. report that PRP contains high concentrations of growth factors such as PDGF, TGF-β, VEGF, and IGF-1, which exert potent anti inflammatory and angiogenic effects that enhance tissue repair and modulate neural pathways involved in arthrogenic muscle inhibition (AMI). By reducing synovial inflammation and improving the biochemical milieu around the reconstructed ACL, PRP may help restore normal afferent input from joint receptors, thereby facilitating better muscle activation patterns and faster strength recovery [25].

Although PRP was administered intra-articularly, its potential influence on periarticular muscle recovery may be mediated by indirect mechanisms. Andia et al. showed that intra-articular PRP can reduce local inflammation and modulate cytokine activity within the joint, which can decrease arthrogenic muscle inhibition—an important factor limiting postoperative muscle activation. Moreover, growth factors released from the synovial environment may exert paracrine effects on periarticular tissues, facilitating neuromuscular recovery [13].

Experimental evidence also supports a neurotrophic mechanism: combined PRP and brain-derived neurotrophic factor (BDNF) treatments have been shown to enhance neural regeneration and improve locomotor performance in animal models [26]. In line with these findings, PRP has been proposed as a modulator of joint neurobiology, contributing to reduced pain and improved proprioception.

These biological mechanisms may partly explain our findings, where participants receiving serial intra-articular PRP injections achieved faster restoration of hamstring strength and improved inter-limb symmetry at 20 weeks.

### 4.4. Limitations

This study has several limitations. First, its retrospective design and relatively small sample size (n = 68) limit causal inference, and the absence of randomization or blinding introduces a risk of selection and performance bias, although groups were comparable at baseline and followed the same rehabilitation protocol. The 20-week follow-up captures only short-term recovery, without information on long-term strength, functional outcomes, or reinjury risk. Additionally, potential confounders such as individual variations in muscle mass, baseline performance, or subtle differences in rehabilitation execution could not be fully controlled.

A further limitation concerns graft type distribution: only a small number of participants received patellar tendon autografts, preventing a reliable analysis of potential PRP-by-graft-type interactions and reducing statistical power for subgroup comparisons. Muscle strength assessments were performed using the Kineo Intelligent Load system rather than a validated isokinetic dynamometer; because reliability metrics (ICC, SEM, MDC) for our specific test protocol are not yet established, the findings should be interpreted as device-specific and may not be directly generalizable to other measurement systems.

Limb dominance was not recorded, which may influence interlimb comparisons, particularly in cases where the injured limb exceeded the contralateral limb in strength. Finally, no imaging or biomarker assessments were available to objectively verify intra-articular or musculotendinous effects of PRP, meaning the proposed biological mechanisms remain hypothetical.

Because this was a retrospective study, an immortal time bias may be present. Although all patients in the PRP group received the injection at the same standardized postoperative time point, there is an inherent period between surgery and the PRP administration during which patients had to remain event-free to be eligible for treatment. This time-related bias should be considered when interpreting the results.

Future research should include randomized controlled trials with larger samples, standardized PRP preparation protocols, validated strength testing procedures, and extended follow-up to determine the long-term clinical relevance of biologic augmentation after ACL reconstruction.

## 5. Conclusions

The experimental rehabilitation protocol incorporating PRP was associated with modest but statistically significant improvements in both primary and secondary outcomes, including enhanced interlimb symmetry (the most clinically relevant parameter), and a trend toward greater strength restoration in the operated limb compared with the contralateral side. Improvements were observed consistently across both isometric and concentric muscle actions, suggesting a potential supportive role of PRP during early postoperative rehabilitation. These superior outcomes were observed exclusively in the group that received PRP, whereas the control group following rehabilitation alone did not demonstrate comparable improvements.

## Figures and Tables

**Table 1:** Baseline characteristics of the PRP and control groups.

Parameter	Group	Mean	SD	Count/Ratio	Mean Difference	*p*-Value
Age (years)	Control	31.3	8.6	—	1.2	0.564
	PRP	30.1	8.6	—		
Sex (m/f)	Control	—	—	25/9	0	1.000
	PRP	—	—	25/9		
BMI (kg/m^2^)	Control	23.8	0.7	—	−1	0.382
	PRP	24.8	0.6	—		
Graft type (hamstring/patellar)	Control	—	—	30/4	−1/1	0.689
	PRP	—	—	31/3		

## Data Availability

The data presented in this study are available on request from the corresponding author.
